# Exploring Students’ Learning Experiences Under the China–Korea Cooperative Teaching Model: A Positive Psychology Perspective

**DOI:** 10.3390/bs15030374

**Published:** 2025-03-16

**Authors:** Lei Song, Zhenzhen Huang, Luhao Cao, Shanshan Yang

**Affiliations:** 1 School of Fine Arts, Central China Normal University, Wuhan 430079, China; 2 Department of Chinese Language Studies, Faculty of Humanities, The Education University of Hong Kong, Hong Kong, China; 3 School of Digital Media Engineering and Humanities, Hunan University of Technology and Business, Changsha 410205, China; 4 School of Foreign Languages, Central China Normal University, Wuhan 430079, China

**Keywords:** learning experiences, learning motivation, positive psychology, PERMA

## Abstract

Based on the theory of learning experiences and the Positive Emotion, Engagement, Relationships, Meaning, and Accomplishment (PERMA) model, this research aims to interpret the learning experiences of students majoring in Animation under the China–Korea cooperative teaching model from a perspective of positive psychology. Purposive sampling was conducted to carry out semi-structured interviews with 25 students and 3 teachers. In the thematic analysis, it was found that students’ learning experiences are accompanied by emotions, both positive and negative, and that emotions are correlated with engagement, relationships, meaning, and achievement, which together constitute students’ learning experiences. In addition, this research confirms that students’ learning motivation and learning experiences mutually affect each other, with learning motivation affecting students’ learning experiences on the one hand, and positive elements of learning experiences enhancing students’ learning motivation and bringing positive learning outcomes on the other.

## 1. Introduction

College students’ learning experiences refers to students’ experiences in engaging themselves in both classrooms and extracurricular activities in their college studies (Astin, 1993, 1999, 2016). Specifically, learning experiences involve instructional quality, the level of teacher–student interaction, student engagement, academic level, and student support services. (Pascarella, 1978, 2001; Pascarella & Terenzini, 2005). For learning experiences in transnational higher education cooperation programs, Hellstén and Prescott (2004) further point out that different cultural environments can affect students’ learning experiences, and students’ progress in learning in the process of Chinese–foreign cooperative education can be revealed by understanding students’ learning experiences. Mok (2018) asserts that when studying learning experiences, researchers must consider the implications of the internationalization of higher education on students’ learning experiences.

According to the theory of learning experiences, students’ learning motivation and learning experiences affect their learning outcomes (Astin, 1993, 1999, 2016; Pascarella, 1978, 2001; Pascarella & Terenzini, 2005). The theory of positive psychology also argues that individuals and their experiences are reflected in environments such as family, school, and society, which have a considerable effect on individuals; hence, a good environmental adaptability is needed to achieve the transformation toward positive human qualities (Seligman, 2019; Deb et al., 2020; Cohen et al., 2023). The theory of positive psychology further explains the positive relationship between individuals’ motivations, attitudes, behaviors, and their sense of well-being (Park et al., 2004; Joseph, 2021; Umucu et al., 2022; Niemiec et al., 2020). Hence, learning experiences are closely related to positive psychology. From the perspective of positive psychology, students’ positive emotions, social relationships, learning engagement, and meaning and accomplishment in learning experiences can be further understood, thereby facilitating a deep interpretation of the inherent relationships between students’ learning motivation, learning experiences, and learning outcomes.

Previous studies on transnational cooperative teaching models were mostly conducted from the aspects of the main actors of teaching, namely teachers, as well as teaching, talent cultivation, and teaching strategies. However, the topic has been rarely studied and analyzed from the perspective of students (Maletina et al., 2015; Taşdemir & Yıldırım, 2017; Sanders-Smith & Dávila, 2024). This study explores students’ learning experiences from the perspective of positive psychology, with a view of explaining what the Chinese–foreign cooperative teaching model means to students’ learning experiences and providing insight into the inherent connections between students’ learning motivation and their learning outcomes. Therefore, studying the Chinese–foreign cooperative teaching model from the perspective of students’ learning experiences takes into account the main actors of teaching and learning to benefit the research of Chinese–foreign cooperative teaching.

## 2. Literature Review

### 2.1. Learning Experiences and Motivation

**Figure 1:**
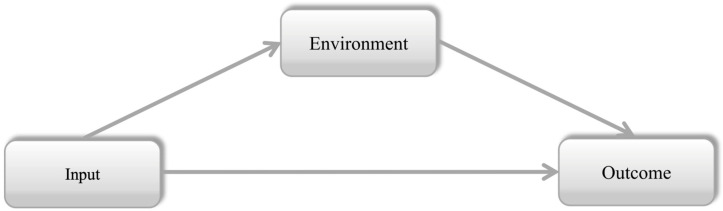
The Input–Environment–Outcome (I-E-O) model. (Astin, 1993).

In the field of higher education, Astin’s (1993) Input–Environment–Outcome (I-E-O) model (Figure 1) stands as a conceptual framework for the systematic evaluation of academic programs and institutional effectiveness. Input refers to students’ initial personal background and learning motivation when they are enrolled in higher education, while environment means students’ experiences in their education, and outcome involves students’ satisfaction and improvements in skills and competences (Astin, 1993). The applications of this model have not only drawn researchers’ attention to students’ outcomes but have also helped them understand students’ learning motivation and the impact of the learning experiences arising from their interactions with environmental factors (Astin, 1993).

In addition, such applications also helped to lay a foundation for researchers to study the relationships between students’ motivation, learning experiences, and future learning outcomes. Astin (1975) asserted that student experience is closely related to the school environment, and positive factors in the school environment promote students’ engagement experience, and vice versa. Since Astin’s I-E-O model was proposed, scholars have been studying the relationship between students’ motivation, learning experiences, and outcomes based on the model (Knight, 1994; Kelly, 1996; Astin & Sax, 1998; House, 2002; Yanto et al., 2011; Duran et al., 2020; Alrizqi et al., 2021; Hudson & Brandenberger, 2022). For example, Hudson and Brandenberger (2022) state that students’ existing attitudes and orientations toward ethical and prosocial issues may determine which types of engagement activities they are attracted to, and how these learning experiences influence their personal development and shift in values.

Building upon his I-E-O model, Astin further proposed the student engagement theory. Student engagement refers to the participation of students in the learning process, both physically and mentally. This theory attaches importance to students’ engagement in all aspects of school activities and emphasizes the role of students’ learning motivation. He pointed out that student engagement involves students’ engagement in teaching, and extracurricular and social activities, and that students’ learning is a process of the students’ engagement and social integration into the campus; students’ final learning outcome is influenced by various aspects of interpersonal communication activities on campus, which has a significant impact on the students’ learning process. The higher the degree of students’ engagement in learning, the better students’ learning outcomes are (Astin, 1999). Learning experiences encompass crucial components such as academic input, teacher–student interaction, and classmate interaction.

### 2.2. Conceptualizing Students’ Learning Experiences from the Positive Psychology Perspective

Positive psychology, as a theory about happiness, identifies positive emotions including joy, serenity, courage, optimism, selflessness, peace, resilience, creativity, and love (Seligman & Csikszentmihalyi, 2000). It focuses on individuals’ positive emotions and long-term, subjective well-being, and emphasizes the research and development of positive human qualities to promote individuals’ growth of well-being and the positive and healthy development of society (Seligman, 2019).

Positive psychology argues that when students’ psychological needs are met, they will be more motivated to learn, feel happier, and be more willing to devote themselves to their studies (Balaguer et al., 2012; Ryan & Deci, 2017; Aelterman et al., 2019). On the contrary, when students’ needs are unfulfilled, they will be less engaged due to negative emotions (Vansteenkiste & Ryan, 2013; Alcaraz et al., 2015; Cohen et al., 2023). Therefore, creating a positive learning atmosphere can stimulate students’ intrinsic motivation, which in turn enhances students’ learning interest and engagement.

Educators can apply the principles of positive psychology to help students establish a positive self-concept and enhance their learning motivation, which is crucial for students to maintain persistence and optimism in the face of challenges. Shoshani and Steinmetz (2014) assert that the interactions between students and teachers, especially the support and encouragement provided by teachers, have a significant impact on students’ learning motivation. Teachers can meet students’ needs by establishing positive teacher–student relationships, creating a learning environment where cooperation and competition coexist, and providing diverse learning activities. Students’ learning motivation and experiences can be promoted by engaging them in the learning process, providing resources and guidance, and creating a classroom atmosphere conducive to learning (Joe et al., 2017). At the same time, communications and interactions among students should not be ignored. A good peer relationship promotes students’ enthusiasm for learning (Lee & Taylor, 2022; Gregersen, 2019).

Seligman (2011) put forward the PERMA theory consisting of the five domains of P = Positive Emotion, E = Engagement, R = Relationships, M = Meaning, A = Accomplishment. All the five domains can be accurately measured and enhanced by learning, and are interrelated. Empirical studies have systematically examined and validated the interrelations among the domains of this framework. First, positive emotions act as drivers of deepened engagement. For instance, research in educational psychology demonstrates that positive affective states significantly enhance learning engagement by fostering sustained attentional focus during academic tasks (Dewaele, 2015; MacIntyre et al., 2019). Second, engagement facilitates self-realization of meaning. For example, engaging in valuable work or learning activities can enhance an individual’s sense of meaning in life (Farmer & Cotter, 2021; Sedeh & Aghaei, 2024). Third, the acquisition of meaning often comes from the interaction of social relationships. Individuals can establish a lasting sense of meaning through the establishment of a sense of social belonging (Lambert et al., 2013; Shoshani et al., 2016). Fourth, relationships can empower achievement. High-quality social relationships provide emotional scaffolding and resource brokerage, optimizing individuals’ capacity for goal attainment through collaborative synergy (Abraham et al., 2021; Thomsen et al., 2023). Relationships can empower achievement. It is believed that high-quality social relationships can provide emotional support for individuals, thereby promoting the accomplishment of achievement (Abraham et al., 2021; Thomsen et al., 2023). Finally, a bidirectional reinforcement cycle exists between achievement and positive emotions. Attainment of goals enhances self-efficacy beliefs and intrinsic motivation, which reciprocally elevate positive affective states through success spirals (Coffey et al., 2016; Sheldon & Lyubomirsky, 2021).

At the same time, the five domains are closely connected with the theory of learning experiences and have a strong explanatory power for students’ learning experiences, both of which argue that students’ input in learning and their relationship with teachers and classmates are related to the meaning and accomplishment of students.

In conclusion, interpreting learning experiences with the positive psychology theory provides a new perspective for the field of transnational cooperative teaching, which helps international educators to promote students’ learning and development more effectively and achieve better learning outcomes. From the perspective of positive psychology, this research aims to explore the learning experiences of college students majoring in Animation under the China–Korea cooperative teaching model (CKCTM), and raises the following research questions:

RQ1: What are the learning experiences of students majoring in Animation under the CKCTM?

RQ2: What are students’ learning experiences from the perspective of positive psychology?

## 3. Methodology

This study is guided by qualitative research methodology in which researchers see themselves as research tools who conduct holistic research on social phenomena under natural circumstances through interviews, observations, field visits, and other methods (Creswell & Poth, 2016; Gall et al., 2017). Qualitative research in the field of education is done by conducting a long-term in-depth exploration of related educational issues by entering schools, classrooms, and other educational venues to fulfill a deep description of the research subjects and examine their internal and external conditions and structural influences (Willis, 2008). The methodology has the following five characteristics: 1. research is conducted in a natural context; 2. in-depth understanding of relevant educational phenomena can be gained; 3. conclusions can be drawn based on original data; 4. results are presented in a textual form; 5. research does not violate ethical codes and focuses on the relationship between the individual and the subjects of the study (Lichtman, 2023).

Interviews are an important part of qualitative research. An interview can be conducted in multiple forms, such as open or semi-open, formal or informal, direct or indirect, and individual or collective, as needed. Interviews in qualitative research are usually conducted using open-ended questions to give interviewees the opportunity to fully express themselves (Creswell & Poth, 2016). This study mainly adopts focus group interviews to collect the interviewees’ views and opinions on the research topic (Morgan et al., 1998).

### 3.1. Participants

This study mainly focuses on senior students and teachers of the Animation major in the largest China–Korea cooperative educational program in Central China. The program is jointly run by the Hubei Institute of Fine Arts in China and Hanseo University in Korea. The word students refers to juniors and seniors majoring in Animation. This group of students was enrolled in 2018 and 2019 through the art college entrance examination (held by the Hubei Institute of Fine Arts) and the National College Entrance Examination, with an age distribution around 20–23 years old. During their first and second years, the students acquire interdisciplinary foundations through cultural courses (art history, 2D animation), while in their third and fourth years they transition to advanced specialization via intensive workshops (3D production, collaborative pipelines) and graduation projects. All participants had completed joint faculty-evaluated training in digital compositing and team-based storytelling before the study, and this cohort uniquely represents learners at the critical juncture between academic training and professional readiness, providing rich insights into learning experiences. Based on purposive sampling, a total of 25 students participated in the interview, and 5 students were included in each group for group interviews.

As the researcher also hoped to learn more information about students’ learning experiences from the perspective of teachers as a triangulation correction of research materials, interviews with teachers were also conducted. The teachers were a Chinese specialty teacher, a Korean specialty teacher, and a Korean language teacher. The three teachers were the head of the Animation major of the China–Korea cooperative program, the lead teacher, and the Korean language teacher from Korea, respectively. All three teachers have studied in China and Korea and were very familiar with the teaching systems of the two universities, creating favorable conditions for the researchers’ in-depth understanding of students’ learning experiences.

### 3.2. Instrument

A semi-structured interview structure was adopted in the study to ensure absolute neutrality in principle. Students could be re-questioned according to their answers and invited to give detailed examples. The interview guide was developed based on Astin’s I-E-O (Input–Environment–Output) model of learning experiences (Astin, 1993), which identifies academic engagement, teacher–student interaction, peer interaction, and learning outcomes as core dimensions. Previous studies grounded in this model have consistently validated these dimensions (Knight, 1994; Kelly, 1996; Astin & Sax, 1998; House, 2002; Yanto et al., 2011; Duran et al., 2020; Alrizqi et al., 2021; Hudson & Brandenberger, 2022; Thibault, 2024), providing a robust foundation for our interview design.

Specifically, the interview guide covers three key modules: academic engagement (e.g., courses learning, animation creation) (Alrizqi et al., 2021), student/teacher interaction (e.g., interaction experiences, class participation) (Thibault, 2024), and learning outcomes (e.g., change and gain, meaning) (Hudson & Brandenberger, 2022). To align with the positive psychology perspective, we incorporated questions emphasizing students’ emotional experiences and psychological transformations, drawing on literature in positive psychology (Seligman, 2011; Shoshani & Steinmetz, 2014). For instance, within the student/teacher interaction module, the questions focus more on the feelings and their psychological impacts. An interview guide for the student and teacher groups was constructed (see Appendix A).

### 3.3. Procedures

Focus group interviews are designed to facilitate student interaction and provide a more comprehensive understanding of students’ learning experiences from various perspectives. The duration of each focus group interview was about one hour, and five interviews were held until saturation was reached (Hennink & Kaiser, 2022), that is, the answers were similar without new data points or topics. For the faculty group, the interview lasted one hour. In addition, group interviews with students and teachers were recorded with their consent and then transcribed after the interviews.

### 3.4. Data Analysis

One of the important approaches to data analysis of qualitative research is thematic analysis for interview data analysis. Thematic analysis emphasizes the accurate positioning and verification of the theme in the data, which consists of stages of in-depth analysis in qualitative research and thematic analysis. Theme analysis and extraction should be carried out on the basis of thematic guidance. This study adopts Boyatzis’ (1998) top-down theme analysis method and identifies and classifies themes according to the five dimensions of the PERMA model proposed by Seligman (2011) and Astin’s I-E-O model (see Table 1). Codes were visualized in the form of images and charts using NVivo to reveal the relationship of codes in a clearer fashion (Azeem et al., 2012). At the same time, the visualization can enhance the reliability of the research and facilitate the sorting of qualitative data, saving researchers’ time (Allsop et al., 2022). Therefore, in this study, NVivo 12 Pro was applied for a three-level encoding of the interview transcripts. In the encoding process, five groups of 25 students were numbered by letters A to Y in the interview transcriptions, and the documents were dated as A (year-month-day) to E (year-month-day); for the teacher part, the documents were labeled as F (year-month-day) for purpose of privacy consideration and document management.

## 4. Findings

The presentation of findings is organized into three thematic modules according to the interview design. By systematically categorizing students’ narratives through these empirically grounded lenses, we aim to provide a comprehensive description of their learning experiences. The subsequent Discussion Section will interpret these findings through Seligman’s (2011) PERMA theoretical framework to uncover latent positive psychological constructs.

### 4.1. Academic Engagement

#### 4.1.1. Changes in Positive Emotions of Engagement in Specialty Courses

Through the coding and analysis of student interviews, it can be found that Chinese and Korean specialty courses have a relatively balanced impact on students’ academic engagement in animation creation. For example, student K (C20211115) reflected that he had gained a lot from the Character Scene Design course provided by the Chinese side, where he could learn how to independently design characters, character images, and scenes. Student X (E20211129) pointed out that he had invested a lot of time and energy into the Joint Assignment course provided by the Chinese side:

*In the Joint Assignment course, I had to stay up late to do homework and achieve various design effects, so I felt rather tired, and other students in the group also put in a lot of effort.*

As for animation creation courses provided by the Korean side, M (C20211115) mentioned that “the 2D Animation Production course provided by the Korean side is full of fun and practicality and gained a great popularity among many students”. The course will first allow students to shoot videos of everyday life scenes and then make 2D stop-motion animations. This course connects animation creation with students’ daily lives, narrowing the distance between animation creation and the social culture in which students live.

In addition to the above analyses from the perspective of student interviews, the teachers also added to students’ learning experiences in relation to their specialty courses and put forward a new point of view. Chinese and Korean specialty teachers and the Korean language teacher (F20211221) all argued that students’ academic engagement is related to their grades. For example, a Chinese teacher (F20211221) pointed out the following:

Students from lower grades have higher levels of curiosity, engagement and interest in specialty courses, leading to a stronger desire to learn. This trend can be maintained until the junior year. But when they are about to graduate in their senior year, they will have all kinds of ideas, some are to prepare for the postgraduate entrance examination, some are to go abroad, some are to find a job, and at the same time, there is the pressure of graduation creation, students tend to slack in their study.

The Korean teachers (F20211221) also held a similar view:

*In the freshman and sophomore years, we have an obvious feeling that these students were very positive and they had a sense of freshness and curiosity. In their junior and senior years, however, it is obvious that their enthusiasm diminished and the time and energy they put into studying were not sufficient.*

#### 4.1.2. Positive and Negative Emotions in Korean Language Learning

Under the CKCTM, mastering Korean language skills is an important prerequisite for smooth communication with and listening to Korean teachers. Hence, students need to learn Korean while studying specialty courses. Based on the coding and analysis of their interviews, it can be found that some students initially showed a certain interest in learning Korean but gradually lost their enthusiasm in the learning process due to some difficulties and the large gap between the language courses taught in school and its practical applications. For example, student E (A20211105) said, “When I first entered the school, I was very interested in learning Korean, but gradually found that the content in the Korean course offered by the school did little to help us fully understand the teaching of specialty Korean teachers, and I still needed to use translation, which caused a vicious circle in my Korean learning. So I gradually reduced my efforts in Korean learning”.

However, some students demonstrated constant interest in learning Korean and successfully improved their Korean level by watching Korean dramas or animations. For example, student O (C20211123) pointed out that “What motivates me to learn Korean is my interest in watching Korean dramas, which can improve my Korean level while entertaining me”.

As mentioned above, the Korean teacher (F20211221) believes that the students’ foreign language learning is similar to their specialty learning, in that they are both motivated and interested in language learning due to their sense of freshness at the beginning of school (Balaguer et al., 2012; Ryan & Deci, 2017; Aelterman et al., 2019) but began to slack off when they started to learn specialty courses in their senior years (Vansteenkiste & Ryan, 2013; Alcaraz et al., 2015; Cohen et al., 2023). In addition, the means by which students learn Korean is not limited to learning on campus, because those who are interested in the language also learn it through the Internet, off-campus training courses, and other methods (A20211105).

### 4.2. Teacher–Student Interaction

#### 4.2.1. Group Differences in Classroom Communication

According to the interviews, the Korean teachers only spend one month in school each semester due to an unbalanced curriculum arrangement between the Chinese and Korean parties, so the time students spent with the Chinese and Korean teachers was unequal, and there were also differences in classroom communication between students and the Chinese and Korean teachers. According to Student M (C20211115), there was a significant amount of communication occurring with the Korean teachers in the classroom:

*The Korean teaching staff communicated with us more often while we were in class. We provided our responses whenever teachers from Korea asked questions. Thanks to the translation provided by the assistant teacher, we could understand them roughly, and the Korean teachers were very enthusiastic, we also actively interacted with them, and would ask questions about the course and our homework.*

The research indicates that the level of Korean language skills influences the dynamics of communication between educators and learners. Student M (C20211115) expressed that his lack of proficiency in Korean hindered his ability to converse with the Korean instructors, relying on translations from the Korean teaching assistants, which made him anxious about engaging with the teachers directly. Q (D20211123) and J (B20211110) noted that “interactions with the Korean educators were restricted to discussions about animation assignments and software usage, and they often sought assistance from the Korean teaching assistants for translation purposes”. The presence of a language barrier significantly impacts the depth of communication and interaction between students and their Korean instructors.

However, teachers believe that student–teacher interaction is similar to the case of students’ academic engagement. First of all, the Chinese teacher (F20211221) argues that “there are three different types of students in the interaction between teachers and students, which are the active, intermediate and passive, with the intermediate type taking up the majority part”. The teacher (F20211221) pointed out the following:

*Engaged learners excel at requesting educators to share their expert insights and guidance regarding their studies. For example, in the graduation creation stage, students usually have two supervisors. Active students consult the opinions of both teachers, and even consult the opinions of other relevant teachers who have given courses to them in the past. The intermediate students, on the other hand, carry out animation creations or communicate about homework according to the requirements of teachers and the deadline set by teachers. Although most students will communicate according to the requirements, they lack initiative. Passive students, on the other hand, need the teacher to urge them before they communicate at a lesser frequency, for less time.*

As such, there are differences in the interaction of different types of students with teachers.

#### 4.2.2. Limitations of After-School Communication

As can be seen from the interviews with students, they have insufficient communication with both Chinese and Korean teachers during their spare time. The majority of the limited after-school communications are academic exchanges, with few concerning daily life. For example, as for the interaction with the Chinese teachers, student P (D20211123) pointed out that “the interaction with Chinese teachers is limited to study, and there is almost no communication about our life”. Student M (C20211115) believed that “the interaction with Chinese teachers is usually limited to the classroom, and the after-school communication only involves some project cooperation”. In addition, another student, H (C20211115), said, “I would keep in touch with teachers that I have a good relationship with after class, sometimes greeting them through social APPs”.

A similar situation also existed in extracurricular communication with the Korean teachers. Most of the interactions mentioned by students were related to study. For example, student M (C20211115) “would give feedback to Korean teachers after class on some questions that were not understood in class”. However, there are also more active students, such as student B (A20211105), who “will take the initiative to care about the daily life of the Korean teachers, such as the food, clothing, housing and transportation”.

According to the analysis of teacher interviews, the Korean teacher (F20211221) pointed out that students would ask some major-related questions that they were interested in or curious about in their spare time, but they were more inclined to ask teachers in a one-on-one manner after rather than in class. He said that, “in daily communication with students, I have the experience of going to the canteen with students occasionally, during which we would talk about some daily life topics”. Meanwhile, the Korean language teacher (F20211221) believes that:

*Similar to the situation of classroom communication, students in the lower grades are willing to ask some questions about Korean learning after class, but only a small number of students in the higher grades carry out communication and interaction after class, and such communications are always limited to a few students.*

### 4.3. Student-Student Interaction

#### 4.3.1. The Myth of Team Collaboration

Team collaboration is an important part of interactions among students in their learning of Animation. When many students talked about the interactive experiences with classmates, the first thing they thought of was team collaboration because it requires joint efforts of different students to complete animation work. However, in the interviews with students, it was found that students’ views vary. Some students thought that the experience of team collaboration was wonderful, while other students considered it to be not as good as expected. Through the analysis of student interview data, this research finds that team collaboration is a “double-edged sword”, and students’ emotions toward it can be either positive or negative. On the one hand, some students hold positive emotions towards team collaboration, just as student C (A20211105) believed:

*Team collaboration enables students to engage themselves in the same thing. In the process of completing the animation work in a group during the 3D Animation Production course in eight weeks, each student is assigned part of the shots and then they would connect the shots into a collective work belonging to all students, which provides a sense of achievement and fun of teamwork.*

Student J (B20211110) also held a positive view of team collaboration. He asserted that “it is a pleasant thing to work together in modeling and creating animation”, and he further pointed out the following:

*In the process of team collaboration, as a team member, I try my best to resolve conflicts and ensure the smooth progress of the animation project. This experience not only taught me how to express my ideas more effectively, but also taught me to listen to others and find consensus. In addition, through constant communication and feedback, I gradually learned how to build trust and enhance team cohesion in the team. The improvement of these communication skills will greatly benefit my personal growth and future career development.*

However, some students have negative feelings towards team collaboration. For example, student G (B20211110) thought that teamwork brings him more pain:

*I have participated in many rounds of teamwork, but in most cases, only a few individuals do the majority part of the work. It was specified in the beginning that everyone should work together in the same direction, but in the end, there is only one person left to worry about the project. That made me frustrated, and do not want to carry out teamwork anymore.*

As mentioned above, although team collaboration has both advantages and disadvantages, there are students experiencing unhappiness, but the final result is satisfactory. Student O (C20211115) mentioned the following:

*I remember very clearly that there was a course called Joint Assignment, in which we worked with all dormitory members to complete an animated short film. In the beginning, there was a fierce debate in the group at a professional level, and everyone had a good idea. Because there were six people’s ideas, everyone’s style was integrated into the scene, and the final scene showed the characteristics of the six people, so this was the most impressive experience of team collaboration for me.*

In addition, most of the student–student interactions mentioned by the teachers focused on classroom team collaboration and academic exchange activities after class. For example, a Chinese teacher (F20211221) pointed out that teamwork in animation majors is a task that takes a lot of time and energy. Students need to work together to complete a creative task that combines script writing, storyboard design, scene drawing, 3D modeling, video editing, and music and sound effects. In the process, there is both division of labor and cooperation as well as mutual assistance and debate. Overall, team collaboration is crucial for animation creation, which can promote students’ mastery and application of professional knowledge and enhance understanding and trust among classmates.

Therefore, although different students have different emotions towards team collaboration, they can still have a relatively pleasant team collaboration experience with a reasonable division of responsibilities (Lee & Taylor, 2022; Gregersen, 2019).

#### 4.3.2. A Lack of Inter-Class or Inter-Specialty Interaction Between Students

The interviews showed that inter-class and inter-specialty interactions among students mostly took place among classmates they are familiar with, and they seldom take the initiative to get to know classmates from other classes or specialty areas. Just as student A (A20211105) mentioned:

*Because we have different specialty orientations, we basically do not discuss with students from a specialty area that is not closely related. In most cases, we mainly chat and interact with students in the same specialty area.*

Students do not have a deep understanding of the knowledge of other specialties, nor do they have an awareness of inter-specialty or interdisciplinary cooperation. As a result, students have less interaction with classmates in other classes or specialties and lack in-depth interactions in these specialty areas.

Some students also pointed out that club activities are a good way to get to know students from other classes or specialties, and some students made good friends when participating in these activities. For example, student X (E20211129) thought that “joining clubs gave me the opportunity to get to know students from different majors and exchange club work-related experience together”. Y (E20211129) said, “In the club, sometimes we would go out for dinner or play out, and then get to know some students from other grades or classes”. However, not all students can get to know more schoolmates in club activities. For example, students H and G (B20211115) thought that “club activities are few and do not provide many opportunities to interact with classmates from other classes and majors”. It can be seen that students’ choice of club, and the organizers of club activities, have a certain impact on students’ communication and interaction with other classes and majors.

### 4.4. Learning Outcome

#### 4.4.1. Specialty Knowledge

For Animation majors, specialty courses take up the largest proportion of hours in their learning. During this process, many students agree on the importance of the acquisition of specialty knowledge. For example, student X (E20211129) confirmed that “a lot of ideas and concepts of animation creation” have been acquired in courses offered by the Chinese side. Student S (D20211123) believed that courses offered by the Chinese side helped them “broaden their artistic vision and mindset, and enable them to use different forms or materials to create works”. T (D20211123) thought that in the courses offered by the Korean side, he “got to know the model of industrial animation production lines such as Disney, and understood to some extent the operation mode of the animation market”. D (A20211105) pointed out that the Korean teacher led the students to start from the early stage of animation production. They have been helping students understand the whole animation production process, providing help even for the subdivisions of their specialty areas since their junior year. In particular, in the interview of the second group, five students (F, G, H, I and J, B20211110) agreed that “they have a high interest in learning Special Effect Production for Videos offered by the Korean side. Although the course requires a large amount of homework, they can learn a lot of specialty knowledge”. Specifically, G (B20211110) mentioned:

*The specialty knowledge taught by Korean teachers is more closely associated with the animation market, which is helpful for our future employment. Therefore, I still like the course very much despite the fast pace of learning.*

According to the analysis of the teacher interview, the Chinese teachers (F20211221) believed that “students relatively hold open ideas for animation creation in this process, they also have their own ideas about the works, and their specialty skills have gradually matured”. On the other hand, a Korean teacher (F20211221) believed that:

*In learning the content taught by Chinese and Korean teachers, students would see different teaching concepts collide amidst multicultural integration, which will be of help for sparking their ideas of animation creation.*

Thus, students can learn different specialty knowledge from both the Chinese and Korean teachers, but these teachers have different emphases. Students gain more theoretical and thinking-related knowledge about animation creation from the Chinese teachers versus more practical knowledge about animation creation from the Korean teachers.

#### 4.4.2. Language Skill

Under the CKCTM, students inevitably have to communicate with the Korean teachers, and the Korean language is also a compulsory course for this specialty area. Improving language skills is also a topic frequently mentioned by students. For example, student A (A20211105) pointed out that under the CKCTM, “I am interested in Korean, so I also signed up for training courses outside the school in addition to the on-campus course, so my Korean language skill has improved a lot”. Student F (B20211110) also mentioned that “I am still taking the Korean test, and my Korean skill has improved in the process”. Student M (C20211115) thought that because of the language barrier in communicating with the Korean teachers in class, “I have been working hard to learn Korean, and I have made progress compared with where I was in the beginning”.

Further interpretation in the interview with the Korean teacher (F20211221):

*As a communicative tool, Korean is still helpful for students’ personal development after their graduation and even after they are employed in society. Compared with those who have no Korean skills or only speak English, those who are proficient in Korean are more competitive. Secondly, for the improvement of their own personal competence, or for their future development, Korean proficiency gives them more chips.*

The implementation of the China–Korea collaborative teaching approach has led to varying enhancements in students’ proficiency in the Korean language, alongside their foundational understanding of animation concepts.

#### 4.4.3. Team Collaborative Ability

Based on the code analysis of student interviews, it can be found that students’ collaborative ability mainly originates from team collaboration in the animation creation process. For example, student J (B20211110) believed that “team collaboration is very helpful to improve our communication skills, because we need to coordinate with each other according to work allocation of various members in that process”. Students B (A20211105) and M (C20211115) also pointed out that they “gained the ability to collaborate with other students to create animation in team collaboration”. A teacher (F20211221) also held a positive view on the improvement of students’ team collaboration in animation creation. Therefore, with a reasonable division of responsibilities, students can still gain different degrees of team collaborative ability in the process of group work.

#### 4.4.4. Aesthetic Quality

The results showed that the students believed that the Chinese teachers lay more emphasis on the cultivation of aesthetic literacy and creativity in their teaching, particularly in the case of the interview with the second group. Some students also share a similar view, as said by student G (B20211110):

*Chinese teachers will give students a broader creative space, pay attention to the aesthetic training of animation creation, fully stimulate students’ imagination and provide more freedom for creation. On the other hand, Korean teachers focus more on the training of technical aspects. They will specify a theme, and ask us to carry out standardized animation production according to the requirements specified in the classroom teaching of the animation production process.*

The student expressed his preference for the cultivation of aesthetic quality. Student J (B20211110), on the other hand, preferred the Korean teaching style, saying that “technology is more practical than aesthetic quality and creativity as it helps turn ideas into reality”. According to the results of the teacher interview, a Chinese teacher (F20211221) also pointed out that “students are exposed to more novel artistic knowledge under the CKCTM, and an international perspective gained by students is also conducive to the improvement of their artistic accomplishment”.

It is evident that students feel the Chinese curriculum prioritizes enhancing their aesthetic abilities, suggesting that their aesthetic development largely arises from this educational framework. On the other hand, the teachers complement the international learning opportunities arising from the CKCTM, which also contributes to the improvement of students’ aesthetic accomplishment and the creativity of animation creation.

#### 4.4.5. Values

In addition to the above learning-related outcomes, students also have considerable feelings about the gains in their outlook on life and values. For example, student T (D20211123) mentioned that “with teachers’ help, I have been able to get a clearer picture about my own path, which made me more adventurous and exploratory in the path of art”. Student G (B20211110) believed that “in the process of learning, I am more clear about my own values, and in the process of creation, I do not need to care about other people’s opinions, and it is more important to establish my own personality”. On the other hand, student H (B20211110) considered:

*Under the influence of the relatively relaxing atmosphere in an institute of fine arts, I have developed a peace of mind compared with the anxiety that I had in the beginning. Now I am able to face difficulties arising from life and study more placidly.*

At the same time, this research also found that students F (B20211110) and J (B20211110) believed that the cultural exchange environment provided by CKCTM helps students understand Korean and international cultures and art and appreciate and tolerate different cultures, as well as forcing them to think about using an intercultural concept to create animation.

The findings from the teachers’ interviews indicate that the collaborative teaching approach between China and Korea positively influences the expansion of students’ perspectives and cognitive abilities. A Korean teacher (F20211221) believed that “students can appreciate different cultures and think about their own goals or views on some events with a more global perspective”. A Chinese teacher (F20211221) further pointed out that:

*Compared with regular animation majors, a higher percentage of the students of this program go abroad for further study, and they have a clearer plan about their study. Many students have already begun to prepare their work portfolios and other materials used in the application process, which is also a reflection of their life planning.*

To sum up, what students gain from their outlook on life and values is, on the one hand, broadening their vision and establishing intercultural values, and on the other hand, adjusting their life and academic planning.

## 5. Discussions

### 5.1. Emotions

The results of this research confirm that students’ learning experiences reflect situations accompanied by positive and negative emotions (Balaguer et al., 2012; Ryan & Deci, 2017; Aelterman et al., 2019; Vansteenkiste & Ryan, 2013; Alcaraz et al., 2015; Cohen et al., 2023). Students experience both positive and negative emotions in their academic engagements, teacher–student interactions, classmate interactions, and learning experiences. On the one hand, it proves that students’ learning experiences are generated by interactions with people, things, and objects in the university environment, among which positive factors will give students pleasant learning experiences (Astin, 1999, 2016); on the other hand, it can also be seen that the positive and negative emotions of students’ learning experiences also affect students’ learning motivation. For example, students’ frustration arising from their encounter with difficulties in Korean learning will lead to a decline in motivation, ultimately affecting the improvement of their Korean skills (Balaguer et al., 2012; Ryan & Deci, 2017; Aelterman et al., 2019; Vansteenkiste & Ryan, 2013; Alcaraz et al., 2015; Cohen et al., 2023), and the level of Korean proficiency affects the learning outcomes of specialty courses. Based on the I-E-O model proposed by Astin (1993), this paper further reveals the internal relationships between learning motivation, learning experiences, and learning outcomes. That is, learning motivation and learning experiences interact with each other. On the one hand, learning motivation affects students’ learning experiences, and the findings of this study further prove that. On the other hand, positive elements of learning experiences enhance students’ learning motivation, and vice versa. Therefore, educators should create a learning environment conducive to learning to promote students’ learning motivation and experiences (Astin, 1993, 1999; Seligman, 2019; Deb et al., 2020; Cohen et al., 2023; Park et al., 2004; Joseph, 2021; Umucu et al., 2022; Niemiec et al., 2020).

### 5.2. Engagement

Students’ engagement in the specialty courses and the Korean language changes with the vanishing sense of freshness. In the subsequent learning process, students’ engagement in Korean decreases due to the difficulties in learning, course content arrangements, and the distraction from specialty courses (Balaguer et al., 2012; Ryan & Deci, 2017; Aelterman et al., 2019; Vansteenkiste & Ryan, 2013; Alcaraz et al., 2015; Cohen et al., 2023). It is apparent that the drive for learning in students is swayed by psychological developments throughout the educational process, with their learning psychology and motivation experiencing dynamic shifts as a result of numerous factors. For example, interesting animation knowledge and practical courses play a positive role in students’ enthusiasm, while the increase in the difficulty of Korean language learning reduces students’ enthusiasm. As for the CKCTM, foreign language learning is of great significance to students, which directly and indirectly affects their communication and learning outcomes with Korean teachers. Therefore, teachers can adopt various strategies to improve students’ enthusiasm for Korean learning. In combination with professional courses, relevant Korean materials and case studies can be used to enable students to understand the practical application value of the language. Teachers should design highly interactive classroom activities, pay attention to the changes in students’ learning motivation, and provide timely feedback and guidance to help them overcome difficulties, and maintain, or even enhance, their learning motivation (Joe et al., 2017).

### 5.3. Relationships

Interpersonal relationships in students’ learning experiences can be divided into interactions with teachers and those with classmates. First, at the level of teacher–student interaction, there are inter-group differences in classroom communication between students and teachers, and the frequency of interaction decreases across the different classifications of active, neutral, and passive-type students. However, there are limitations in after-school communication, most of which focus on academic communication, and few concern everyday life experiences.

Additionally, at the level of classmate interaction, the classroom interaction among students mostly focuses on team collaboration in animation learning, while some students do not have many opportunities to contact classmates from different classes or specialty areas, and rarely think of interdisciplinary and inter-specialty cooperation, resulting in a lack of inter-class and inter-specialty interactions. One can observe that the experiences gained through learning are closely intertwined with the relationship between teaching and learning. Since animation creation is a complex task involving different processes and information gaps, it requires team members to cooperate and exert their team strength to complete the task. Meanwhile, team members can give full play to their strengths in dubbing, editing, and other teamwork tasks. Therefore, a good relationship between teachers, students, and classmates is conducive to the students’ learning on the Animation major and their sense of belonging (Joe et al., 2017; Lee & Taylor, 2022; Gregersen, 2019).

### 5.4. Meaning

Students’ learning experiences are also very important for shaping their outlook on life and values. On the one hand, these experiences broaden their vision, and on the other hand, the experiences also adjust their life and academic plans. Under the CKCTM, students are able to open up their vision, understand the diversity of animation creation, and gain intercultural knowledge.

At the same time, the CKCTM also has a certain impact on students’ college plans, especially in terms of promoting their desire to study abroad to broaden their horizons. Under this pedagogical framework, students not only encounter diverse educational practices from around the world but also develop an appreciation for cultural variety, which they can leverage to innovate in animation through intercultural thinking. In addition, by participating in international exchange programs, students are encouraged to think about their career and study path from an international perspective and get ready for their future development (Hudson & Brandenberger, 2022; Seligman, 2019).

### 5.5. Accomplishment

The students have also achieved a lot under the CKCTM, in terms of specialty knowledge, Korean language skills, team collaboration, and aesthetic accomplishment. At the same time, various achievements of students further stimulate their learning motivation to deepen their studies (Balaguer et al., 2012; Ryan & Deci, 2017; Aelterman et al., 2019).

Initially, the contrasting educational approaches in China and Korea have resulted in variations in how students acquire specialized knowledge in their respective courses. However, it is evident that Chinese educators focus on establishing a solid theoretical base and fostering creative thinking for animation projects, whereas their Korean counterparts emphasize helping students grasp the workings of the animation industry and the production process. Due to the difference in teaching emphases, most of the students’ academic engagement in software operation is derived from the Korean teaching, helping them gain a certain degree of animation software skills in the process.

Second, it can be found from the research that students mainly learn Korean as a foreign language, and they have some problems in that process, for example, the on-campus Korean which is taught cannot meet the needs of some students, forcing them to turn to other channels to learn the language. However, in general, students can improve their Korean skills to some extent.

At the same time, students have a lot of team collaboration when learning about, and creating, animation. Although different students hold different emotions about team collaboration, a reasonable division of responsibilities helps to promote the improvement of students’ team collaboration. Teachers should give certain guidance in the division of responsibilities in team collaboration to help students find their own roles, optimize and improve students’ interaction experience with their classmates, and improve students’ team collaboration.

In addition to the acquisition of specialty and technical knowledge on animation, students’ improvement of aesthetic quality in learning is also an important part to support the overall artistic level of animation creation, especially in the level of creativity and aesthetic taste related to visual arts. It is evident that the experiences students encounter during their learning process foster development in several areas (Park et al., 2004; Seligman, 2019; Joseph, 2021; Umucu et al., 2022; Niemiec et al., 2020).

In summary, students’ learning experiences indicate that there are still many aspects that need optimization. This study proposes the following ideal practice framework. Firstly, course arrangement: The Chinese and Korean course architectures should establish complementary specializations to avoid homogenization, dynamically adapting to evolving student competencies and the demands of the animation industry. For example, the Chinese curriculum should update the animation aesthetic shaping course in line with the development and changes of the times, while the Korean curriculum should introduce cutting-edge technology into the course according to market trends, to help students understand the latest animation market trends and production processes. Secondly, Korean language teaching: The selection and formulation of Korean teaching materials should be in line with the needs of animation majors. In addition to basic Korean learning, courses covering animation-specific terminology should be included to help students adapt to their specialty courses. Furthermore, extracurricular language immersion initiatives, such as campus conversation hubs and dedicated language resource centers, should be implemented to provide contextualized linguistic practice. Thirdly, teaching strategy: Interdisciplinary teaching methodologies that encourage collaboration between animation students and complementary disciplines should be adopted. Conventional cooperative teaching models should be transcended by institutionalizing teacher–student co-creation mechanisms to deepen engagement in the learning process. Fourthly, interaction and collaboration: academic seminars and cultural exchange activities should be implemented to foster intensive relationship-building. Additionally, tripartite dialog forums enabling student–teacher idea exchange should be established, prioritizing bidirectional feedback loops over unilateral consultation. These strategies directly address the identified challenges, ensuring a more cohesive educational process that bridges cultural and disciplinary divides.

## 6. Implications and Conclusions

From the perspective of positive psychology, this study presents an in-depth interpretation of the learning experiences of animation majors under the CKCTM. The results of this research show that students’ learning experiences cover all five dimensions of the PERMA model, namely, emotion, engagement, relationships, meaning, and accomplishment, and are concretely reflected in aspects of students’ academic engagement, teacher–student interactions, classmate interactions, and learning outcomes. At the same time, it was observed in the study that the interrelation between the five dimensions is consistent with the previous research. Specifically, the positive emotions experienced by students can promote their enthusiasm for academic studies. Also, students can further establish their outlook on life and values by participating in various learning activities and interacting with teachers and classmates. In the teamwork of the animation course, students can experience a sense of accomplishment, which can further enhance their enthusiasm for learning. According to the analysis, although the PERMA model can better interpret students’ learning experiences, its interpretation is not comprehensive. The model fails to fully consider the role of negative emotions in the learning process. In fact, students’ negative emotions also affect learning motivation, which in turn affects learning outcomes. In addition, the study also shows that freshness is of great importance in stimulating students’ enthusiasm for learning during the learning process. As students’ progress from lower grades to high, their enthusiasm for learning gradually fades and decreases.

**Figure 2:**
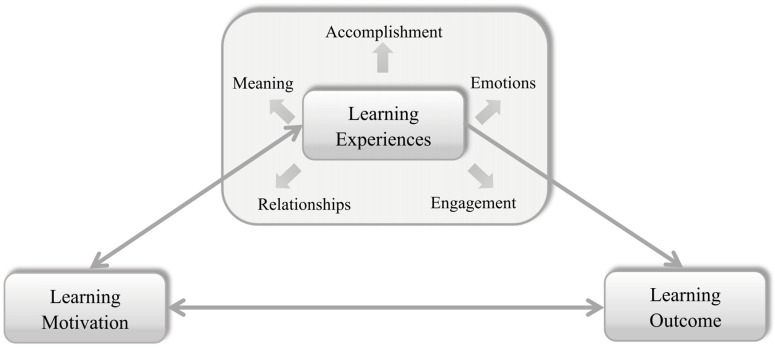
The visual representation of the relationship between learning motivation, experiences and outcome.

In summary, this study, based on the theoretical foundation of Astin’s I-E-O model and Seligman’s (2011) PERMA model, verified the correlation between the two. Compared with most previous studies on learning experience, which obtained an overall overview through large-scale quantitative surveys (Knight, 1994; Kelly, 1996; Astin & Sax, 1998; House, 2002; Yanto et al., 2011; Duran et al., 2020), this study deeply explains the learning experience of students under the CKCTM framework from the perspective of positive psychology, and provides a new perspective for the study of the learning experience of students in the context of transnational higher education. The study clarified that students’ learning motivation and learning experiences mutually affect each other. Positive learning motivation brings positive learning experiences; similarly, pleasant learning experiences stimulate students’ learning motivation and bring positive learning outcomes. Positive learning motivation brings positive learning experiences; similarly, pleasant learning experiences further stimulate students’ learning motivation and bring positive learning outcomes (Figure 2).

For animation students, on the one hand, students gain specialty knowledge and skills, such as animation software operation, visual presentation, character scene design, etc., while on the other hand, they also improve their aesthetic quality, intercultural understanding, and Korean language ability. In addition, they gain team collaboration abilities in the process of interacting with classmates, and establish a life view and values with interaction with teachers.

The presentation of the results here does not mean the end of the study. On the contrary, the results help lay a solid foundation for subsequent extended research. Since the subjects of this study are limited in this specific project, subjects of future research can be expanded by combining the Astin’s (1993) I-E-O model and Seligman’s (2011) PERMA model with more quantitative tools, thereby exploring students’ learning experiences under the mode of transnational education cooperation in a more comprehensive way.

## Figures and Tables

**Table 1:** Repertory grid for emerging the themes.

Themes	Sub-Themes	Significant Statements
Emotions	Positive emotions	We have an obvious feeling that these students were very positive and they had a sense of freshness and curiosity. (F20211221)
	negative emotions	That made me frustrated, and do not want to carry out teamwork anymore. (B20211115)
Engagement	Specialty courses study	The 2D Animation Production course provided by the Korean side is full of fun and practicality and gained a great popularity among many students. (C20211115)
	Korean language learning	What motivates me to learn Korean is my interest in watching Korean dramas, which can improve my Korean level while entertaining me. (C20211123)
Relationships	Teacher–student interaction	The Korean teachers were very enthusiastic, we also actively interacted with them. (C20211115)
	Student-student interaction	Each student is assigned part of the shots and then they would connect the shots into a collective work belonging to all students, which provides a sense of achievement and fun of teamwork. (A20211105)
Meaning	Values	I have been able to get a clearer picture about my own path, which made me more adventurous and exploratory in the path of art. (D20211123)
Accomplishment	Specialty knowledge	The specialty knowledge taught by Korean teachers is more closely associated with the animation market, which is helpful for our future employment. (B20211110)
	Korean language skill	As a communicative tool, Korean is still helpful for students’ personal development after their graduation and even after they are employed in society. (F20211221)
	Team collaborative ability	Team collaboration is very helpful to improve our communication skills. (B20211110)
	Aesthetic Quality	Chinese teachers will give students a broader creative space, pay attention to the aesthetic training of animation creation. (B20211110)

## Data Availability

The datasets generated and analyzed during the current study are not publicly available due to the inclusion of private information and the extent of the informed consent provided by the participants but are available from the corresponding author upon reasonable request.

## Abbreviations

The following abbreviations are used in this manuscript:

CKCTM	China–Korea cooperative teaching model
I-E-O	Input-Environment-Outcome
PERMA	Positive Emotion, Engagement, Relationships, Meaning, and Accomplishment

## Appendix A

### Appendix A.1

Student section

1. Academic engagement:
  - Which courses do you prefer? How did you feel during the course?
  - Can you share the most impressive experience you have in animation creation?
2. Student/Teacher interaction:
  - What are the most impressive student interaction experiences that you can share with us?
  - How about interacting with students from different classes or majors? How did you feel about it?
  - How did you interact with Chinese and Korean teachers? How do you feel about them?
  - From the perspective of teaching style, what influence do you think the Chinese and Korean teachers have on your specialty study?
3. Learning outcome
  - What do you think is the biggest change and gain in the learning process of CKCTM?
  - What’s its meaning?

### Appendix A.2

Teacher section

1. Academic engagement:
  - How engaged do you think students are in their studies?
  - Change in attitude?
2. Classmate/teacher/student interaction:
  - How would you describe your interaction experience with students?
  - How do you feel about it?
3. Learning outcome
  - How do you think the collaboration in teaching between China and Korea impacts students’ expertise in their subjects and aids in their personal development?
